# Commentary: Efficacy and safety of adding immune checkpoint inhibitors to standard chemotherapy or chemoradiotherapy for advanced or recurrent cervical cancer: a meta-analysis

**DOI:** 10.3389/fimmu.2026.1902016

**Published:** 2026-08-03

**Authors:** Hui Liu, Zhe Zhang, Jiabin Xu, Junjie Li

**Affiliations:** 1 Department of Obstetrics, Zibo Central Hospital, Zibo, Shandong, China; 2 Department of Gynecology, Zibo Central Hospital, Zibo, Shandong, China; 3 Department of Anesthesiology, Zibo Central Hospital, Zibo, Shandong, China

**Keywords:** meta-analysis, methodological comments, publication bias detection, random-effects model, randomized controlled trial

We have read with considerable interest the recently published meta-analysis by Le Zhou et al. in Frontiers in Immunology, which synthesized available randomized controlled trial (RCT) evidence to evaluate the efficacy and safety profile of immune checkpoint inhibitors (ICIs) combined with conventional chemotherapy or chemoradiotherapy for advanced and recurrent cervical carcinoma (1). This study addresses an important and clinically relevant question in gynecologic oncology, particularly in the context of rapidly evolving immunotherapeutic strategies. By integrating data from multiple clinical trials, the authors provide valuable evidence regarding the potential role of ICIs combined with conventional chemotherapy or chemoradiotherapy in patients with limited treatment options. The findings may contribute to future individualized treatment decisions and provide meaningful insights into the clinical application of immunotherapy in cervical cancer. This is particularly relevant in evolving immunotherapy. Given the increasing integration of immunotherapy into cervical cancer treatment strategies, rigorous evidence synthesis is essential to ensure that clinical recommendations are supported by reliable and appropriately interpreted data. Methodological refinement therefore remains important for improving the applicability of meta-analytic findings.

Nevertheless, several methodological aspects of the current meta-analysis merit further consideration. These concerns do not diminish the clinical importance of the study, but addressing them may help improve the robustness and interpretability of the pooled estimates (2). These methodological considerations relate to the selection of statistical models, the interpretation of publication bias assessments using Egger’s regression and funnel plots, and the evaluation of trial quality based solely on the modified Jadad scale. These methodological considerations may affect the precision and interpretation of pooled effect estimates and may influence the robustness of the authors’ clinical conclusions; we present balanced, constructive commentary on each issue, alongside pragmatic revisions aligned with widely accepted evidence synthesis practice.

## Statistical model selection for meta-analysis

The original authors selected fixed-effect pooling based on an I² threshold below 50%, a practice that may be reasonable in certain contexts but warrants further methodological consideration rather than rigid dismissal. The Cochrane Handbook Chapter 10 clarifies that the choice of meta-analytic model is ideally informed by pre-specified considerations in the review protocol, including anticipated clinical and methodological heterogeneity across trials, rather than relying solely on *post-hoc* heterogeneity statistics such as I² or Cochran’s Q (3). However, methodologists recognise legitimate flexibility in this choice, contingent on review objectives and underlying assumptions of treatment effect distribution.

Fixed-effect models operate under the assumption that all included trials estimate a common underlying treatment effect, an assumption most plausible when trials share similar patient inclusion criteria, matched ICIs, consistent chemotherapy backbones, uniform radiotherapy schedules and comparable disease stages. The present meta-analysis pools heterogeneous RCTs incorporating distinct anti-PD-1/PD-L1 agents, treatment-naive locally advanced cohorts and heavily pretreated recurrent metastatic populations, with variable radiotherapy integration. Such clinical diversity may indicate the presence of inter-study variation, making random-effects pooling a common default to accommodate potential differences in true treatment effects.

We acknowledge that fixed-effect models remain defensible in narrow, highly homogeneous datasets. Still, selecting the analytical model primarily according to observed I² values carries limitations: it may narrow confidence intervals and potentially affect the certainty of estimated survival and anti-tumour benefits from ICIs. For balance, we suggest that the original authors consider re-analyzing primary endpoints using random-effects models as a complementary or sensitivity framework, while briefly discussing fixed-effect outputs as a secondary sensitivity comparison to illustrate how model choice shapes interpretive range. Such additional analyses would help clarify the robustness of the findings across different statistical assumptions and provide readers with a more comprehensive understanding of the evidence.

## Assessment of publication bias with limited trial numbers

The original meta-analysis included only five eligible RCTs for quantitative synthesis. Cochrane guidance notes regression-based publication bias tests (Egger’s and Begg’s tests) and formal trim-and-fill correction have limited statistical power with fewer than 10 studies, which may increase the possibility of misleading interpretations of asymmetry signals rather than providing reliable evidence of publication bias (2). Small trial counts mean funnel plot visual asymmetry can stem from clinical heterogeneity, variable trial quality or uneven baseline patient risk rather than unpublished negative results alone.

Our commentary does not argue that publication bias cannot be evaluated entirely with five trials; instead, all quantitative test outputs require careful interpretation. The original manuscript’s statistically significant Egger’s P-values for objective response rate (ORR) and grade 3–5 adverse events, paired with subsequent trim-and-fill adjustment, should be interpreted cautiously given the limited statistical power of these approaches in small meta-analyses. Consistent with Cochrane recommendations, we suggest that the authors consider replacing formal Egger/Begg’s quantitative testing and trim-and-fill correction with a qualitative narrative discussion acknowledging inherent limits to small-study publication bias evaluation. Readers should be reminded that any funnel plot asymmetry observed in this limited sample should not be interpreted as definitive evidence of missing unpublished trials.

## Risk-of-bias evaluation tools

The original authors relied exclusively on the modified Jadad scale to assess RCT methodological quality. Current Cochrane and GRADE methodological frameworks recommend greater emphasis on the Cochrane Risk of Bias Tool 2.0 (RoB2) for domain-specific bias appraisal, yet we recognise the Jadad scale retains widespread historical usage in older meta-analyses. The modified Jadad scale evaluates four domains, including random sequence generation, allocation concealment, blinding and dropout reporting, but *does not directly address several additional domains emphasized in contemporary risk-of-bias assessment*, including deviations from intended interventions, missing outcome data and selective reporting.

Aggregated Jadad scoring creates offset bias, whereby severe flaws in one domain may be partially masked by strengths in other domains, potentially resulting in oversimplified classifications of overall study quality. GRADE evidence certainty ratings also require granular RoB2 domain judgements rather than composite Jadad summary scores for bias-related downgrading. We recommend supplementing full RoB2 assessments across all five standard bias domains for every included trial, while briefly contextualising Jadad’s historical prevalence to frame this tool transition as an evolution toward contemporary best practices rather than a rejection of previous methodological approaches.

We fully acknowledge the valuable clinical contribution of this meta-analysis exploring ICI combination therapy for advanced cervical cancer, an unmet clinical need in global gynecologic oncology practice. To further enhance the methodological transparency and clinical interpretability of this important work in accordance with contemporary evidence synthesis recommendations, we respectfully propose three targeted revisions: consider re-analyzing efficacy and safety endpoints using random-effects meta-analysis as an additional analytical framework; interpret Egger’s/Begg’s quantitative publication bias assessments and trim-and-fill analyses with greater caution in view of the limited number of included trials, with emphasis on qualitative discussion of small-study effects; and supplement the current risk-of-bias evaluation with RoB2 assessments rather than relying solely on modified Jadad scoring. We hope these constructive methodological comments could assist the authors to refine their valuable research and optimize the reporting quality of this cervical cancer immunotherapy meta-analysis.
